# Negative Data, Oh No What Should I Do? How Publication of Negative Data Removes Roadblocks to Productive Research from the Perspective of Scientists Who Perform the Experiments

**DOI:** 10.4049/immunohorizons.2300032

**Published:** 2023-05-30

**Authors:** Angela K. Beltrame, Johnathon J. Caldon, Cody J. Gurski, Zivar Hajiyeva, Nathan J. Meinhardt, Kelli C. Sommers, Savannah D. Neu, Bonnie N. Dittel

**Affiliations:** * Versiti Blood Research Institute, Milwaukee, WI; † Department of Microbiology and Immunology, Medical College of Wisconsin, Milwaukee, WI

We have all been there: you developed a provocative, compelling hypothesis and then spent days, months, and even years generating the data to test it, and your end results are negative. This is the curse of trainees because they have shown their scientific muster by reading the literature, learning and developing new techniques, and very often (in immunology) spending a considerable amount of time generating and breeding an incredibly special mouse line. All for what?

Your data did not support your hypothesis, and you cannot publish your findings because no high-profile journal will even review it. Yes, you develop a new hypothesis and move forward, but you also really want recognition for all your hard work. Alas, you found a solution after reading an article in *ImmunoHorizons* and came to the realization that this is a journal that publishes all high-quality immunological studies, even those that disprove a hypothesis or used mice that do not show a phenotype. You approach your mentor with the idea and with the quick editorial and review timelines of *ImmunoHorizons*, you have a publication. You celebrate and start working on that new hypothesis.

The advantages of publishing in *ImmunoHorizons* are that you will not be given a lengthy list of new experiments to perform, online publishing is quick, the fully open access means your article can be read by anyone, you own the copyright, and it is indexed in Medline. So, the next time your studies are well conducted and are of interest to the immunology community but are not going to win you the Nobel Prize, consider publishing in *ImmunoHorizons*. If you do not believe me, Bonnie Dittel, Ph.D., *ImmunoHorizons* Senior Editor, perhaps the following comments from my laboratory staff and trainees will change your mind.

## A graduate student’s point of view: The positives of negative data

## Angela K. Beltrame, third-year Ph.D. student

I believe that the science community should embrace negative data and make it more available. Being a graduate student, it is daunting to determine what hypotheses to explore due to our limited experience. Therefore, we rely on what we can find in the literature to help shape our experimental design. However, because negative data are not consistently published, there is a real possibility that an experimental question we decide to tackle has already been investigated by another laboratory and negative results were never published. This could cause us to repeat the experiments unknowingly, wasting our time and preventing progress. In addition, we are constantly reminded in graduate school to follow the data, not to hope for specific results, and to remain unbiased. Success and failure are important to personal development as a graduate student. By having a journal that celebrates negative data, we can still be successful and grow as scientists when an experiment yields negative results. This will be essential for our careers and help future researchers to not repeat the same experimental hypothesis.

## Negative data, positive progress

## Johnathon J. Caldon, second-year Ph.D. student

Scientific progress is not always made quickly, and there tends to be a lot of negative data generated in the process of testing a new hypothesis. This is particularly true if you are a first- or second-year graduate student with a lack of laboratory experience compared with your peers. It is therefore valuable for students such as myself to be able to publish negative data because we can build our résumés without a breakthrough in our respective fields, something that is difficult to achieve due to the aforementioned lack of experience. Moreover, a current project of mine will focus on epidemiological risk factors in Black individuals. This kind of research is currently lacking, and it will put more focus on this underrepresented population even if we produce only negative data. However, without more journals to publish that negative data in, this research disparity will be much more difficult to reduce.

## A tale of two peroxidases

## Cody J. Gurski, Senior Research Technician and Lab Manager

We were interested in discerning which mouse macrophage populations expresses myeloperoxidase (MPO) to clarify the scant literature regarding expression of MPO in macrophages and to potentially use MPO to identify macrophage subsets with inflammatory capacity. We characterized monocytes/macrophages in the spleen, small and large intestine, mesenteric lymph nodes, and blood using a variety of cell-surface markers and MPO by flow cytometry (1). Neutrophils, known to express high MPO protein levels, were identified in CD11b^+^-gated mononuclear cells as Ly6G^+^ (2). Neutrophils served as the positive control and were excluded from the monocyte/macrophage analysis. Initially, we were excited to discover that Ly6C^lo^ monocytes/macrophages in every tissue, which are heralded to be mostly anti-inflammatory in nature, in fact expressed MPO at a level considerably higher than that of neutrophils, implying a quite different inflammatory profile for these cells than what was reported in the literature (3, 4). We back-gated these MPO^hi^ cells to better understand their phenotype and found them to be exclusively high in side scatter, a designation sometimes reserved for eosinophils in previously published mouse myeloid cell panels (5). We restained the tissue macrophages, including the eosinophil exclusive marker SiglecF (6). Indeed, the entire Ly6C^lo^MPO^hi^ population in every tissue examined was SiglecF^+^. We considered that it would still be interesting if eosinophils were expressing MPO, a finding that was not yet published in the literature. What we found in the eosinophil literature is that they express their own peroxidase called eosinophil peroxidase (EPO), which shares >70% homology with MPO (7, 8). In all likelihood, the commercial anti-MPO Ab used was cross-reactive with EPO. Although these findings were certainly not as exciting as the anticipated former findings, they are pertinent because at the time there were four commercially available Abs specific to MPO, of which none of the product sheets made mention of EPO or guaranteed exclusive binding to MPO. We inferred that these negative data would be of interest to anyone studying MPO because of the ubiquitous presence of eosinophils in all tissues examined. If eosinophils are not explicitly depleted or otherwise excluded, data obtained while staining for MPO will have a high false-positive staining level in flow cytometry and immunohistology, at least with some commercial Abs.

## Why be thankful that your hypothesis was (un)supported: Clinical insight

## Savannah D. Neu, Ph.D., postdoctoral fellow

We often take for granted the efficacy of medicine without considering off-target effects, especially if they are not printed on the packaging. In the pharmaceutical market, potential adverse events of therapies are listed ad nauseum as they are perceived in the immediacy of clinical trials. These side effects range from minor to severe and common to rare, but they are concatenated together in the shortest paragraphs possible for the sake of legally informing doctors and patients. However, long-term consequences of drugs on the biology of the body are not always understood (or extrapolated) because these consequences may emerge beyond the observation window of clinical trials. Further, many medications are taken in excess of decades, and although off-target changes may not be observed in the short term, they may gradually accumulate or worsen over extended periods. This is a story about studying such long-term treatment impacts.

During my time as a graduate student, our laboratory was investigating B cell depletion therapies (anti-CD20), which are a class of drugs used to treat illnesses, including non-Hodgkin’s lymphoma and multiple sclerosis. Although the reason for efficacy in some disease contexts is not fully understood, the mechanism of action for B cell depletion is explicit. These drugs indiscriminately deplete B cells, which broadly affects all regions of the body.

This raised a red flag in my mind as I understood that not all B cells are bad players. In fact, certain B cells are important for health by keeping our mucosal barriers, like the gut, protected. Concerned by this potential off-target effect of B cell depletion therapy, I gloomily hypothesized that continuous use of these drugs would be harmful for gut health. Our research project was unique for we used a novel dosing strategy to simulate long-term drug administration in mice: an experimental situation and timeline that better reflected human therapy than projects of the past. After years combing through facets of gut function, we concluded that B cell depletion therapies have limited impacts on B cell regulation in the intestines. In other words, these drugs were not harmful to the gut (Neu and Dittel, unpublished observations).

Although arguably not as exciting as discovering new cures for disease, the outcomes of our research are still gratifying because they bolster the perceived safety of B cell depletion therapies. We believe that our results are beneficial for both clinicians and pharmaceutical manufacturers alike, because this work can be used as a resource for how B cell depletion impacts on health. Overall, our negative data should be made accessible and in a meaningful manner.

## The “bedside” side of research

## Zivar Hajiyeva, M.D., research fellow

As we already know, the “bench-to-bedside” approach has made so many valuable therapy options possible. The ultimate goal of both scientists and clinicians is to help patients deal with their diseases in the most efficient way. Being a neurologist myself, I understand all the responsibility of choosing and discussing the appropriate therapy options with my patients. When doing so, I rely on the published data. The evidence shows, however, that half of the completed clinical trials are never published or are published partially (9). As a physician, I understand that I am “the eyes” of my patients when it comes to searching the literature, analyzing the research data, and ultimately suggesting the best possible therapy options to them. I often wished I had access to the “whole picture” of both the positive and negative data to make the best possible health care decisions. In addition, knowing the most specific details helps us when choosing between alternative therapies. I believe the approach of publishing both positive and negative data would benefit both physicians and patients.

## Wrong answers can be right to publish

## Kelli C. Sommers, fourth-year M.D., Ph.D. student

**FIGURE 1.**
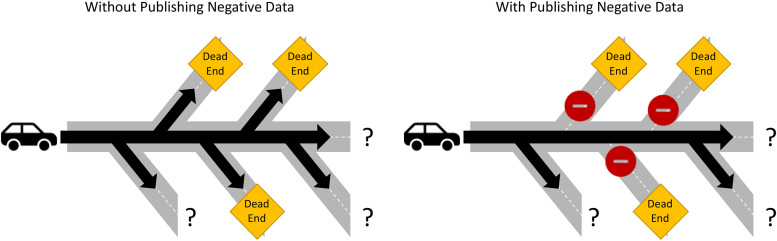
Publication of negative data removes roadblocks that lead to a scientific dead end. This schematic shows the possible routes of a new scientist approaching a research question with and without the publication of negative data. Without the negative data, the roads to known dead ends are still open, potentially causing that scientist to turn off course. Alternatively, with publishing negative data, those roads can be closed, allowing the scientist to direct their travel to new roads with unknown answers.

Finding out an answer, any answer, is worth reporting and provides important information to a field. If only positive findings make it to print, scientific knowledge is cut in half. Publishing negative data provides what does not work or is not involved, and in turn saves time and resources for future studies from pursuing questions whose answers are already known. This is especially important for new scientists entering graduate training who are trying to find their niche for dissertation projects. The first step as they start to investigate is to search the literature for what is known about a particular topic and formulate a question. Without negative data, they discover only half of the story, potentially leading them to the same negative data that were never published (Fig. 1). This wastes both the time and the talent of the individual and prevents them from making real progress in their field. In addition, because publication is often a requirement for graduation from graduate training, accepting papers only on positive data may encourage a mindset within students focused on chasing statistical significance, potentially leading to bad science or biased analysis. By instead recognizing the importance of negative data, the pressure for significance is alleviated, and the focus can shift to thoughtful experimental design and analysis to truly answer the question at hand. Distributing negative data emphasizes the importance of iterative knowledge for a field, building off good science to learn more, both what works and what does not, to progress toward answering the overarching questions within a discipline.

## Final Thoughts

Those are the voices and thoughts of my current trainees. They all work on exciting projects and have lots of great data, but that will not be the case for every line of research. In the latter case, we will summarize the data, proud that they are expertly executed in a well-written manuscript, and submit it to *ImmunoHorizons*. Once accepted, the laboratory will celebrate and begin work on the next great hypothesis. Data are data, which is valuable irrespective of the hypothesis they support or refute.
